# Postinfantile Giant Cell Hepatitis: An Etiological and Prognostic Perspective

**DOI:** 10.1155/2013/601290

**Published:** 2013-03-11

**Authors:** Chhagan Bihari, Archana Rastogi, Shiv Kumar Sarin

**Affiliations:** ^1^Department of Pathology, Institute of Liver and Biliary Sciences (ILBS), D-1 Vasant Kunj, New Delhi 110070, India; ^2^Department of Hepatology, Institute of Liver and Biliary Sciences (ILBS), New Delhi 110070, India

## Abstract

Giant cell hepatitis is common manifestation in pediatric liver diseases, but quite uncommon in adults, only about 100 cases reported in the English literature in the last two decades. Data for the present review were identified by a structured PubMed/MEDLINE search from 1963 to December 2012, using keywords postinfantile giant cell hepatitis (PIGCH), adult giant cell hepatitis, and syncytial giant cell hepatitis in adults and liver. We report a case of postinfantile giant cell hepatitis along with the review related to the etiology and respective outcome, as the literature in the last 20 years suggests. This condition is probably due to idiosyncratic or cytopathic response of individual to various hepatocytic stimuli. It is purely a histomorphological diagnosis and does not establish the etiology. Autoimmune liver diseases are most common etiology, in around 40% of cases, but various viruses, drugs, posttransplant condition, and other causes also have been reported. Prognosis depends upon the etiology. In this paper, we emphasized various causative factors of PIGCH and their respective outcome in patients affected by them. We also highlighted the possible pathogenesis and histopathological spectrum of this entity on the basis of description given in various studies and our limited experience of few cases.

## 1. Introduction

Giant cell hepatitis is a condition characterized by inflammation and large multinucleated hepatocytes in the hepatic parenchyma. Giant cell transformation of hepatocytes along with extramedullary hematopoiesis is a common response in the newborn liver diseases [1–4]. Postinfantile giant cell hepatitis is a rare disorder. It is an unusual regenerative or degenerative hepatocytes response to various noxious stimuli, characterized by the presence of multinucleated cells in liver with generally dismal clinical outcome [1–4]. We report a case of postinfantile hepatitis with review of the literature regarding various etiological agents and their respective prognostic outcome.

## 2. Methods 

Postinfantile giant cell hepatitis (PIGCH) is defined as acute or chronic hepatitis in adults with extensive hepatocyte multinucleation. These cases can be heterogeneous in terms of their clinical, serological, and histological features [1, 3]. PIGCH is purely a histological diagnosis which is based on morphological criteria of conspicuous presence of giant cell hepatocytes; therefore, it is a descriptive term and does not speak about the etiology in any individual case [1]. Facts for the present paper were collected from the structured PUBMED/MEDLINE search from 1963 to 2012. The search was carried out by combining the keywords postinfantile giant cell hepatitis, adult giant cell hepatitis, and syncytial giant cell hepatitis in adults and liver. We have comprehensively categorized the prognostic outcome of various studies into poor, moderate, and good prognosis groups. In poor prognostic group, we have included the patients who had acute liver failure, acute decompensation on chronic liver disease, and death with due diagnosis of this entity. In moderate prognosis group we have put those patients who had rapid onset of cirrhosis following the diagnosis of PIGCH, and in good prognosis category, we have grouped those patients who had mild hepatitis. Here, we report a case of postinfantile giant cell hepatitis that had acute liver failure and undergone living donor related liver transplant.

## 3. Report

**Figure 1:**
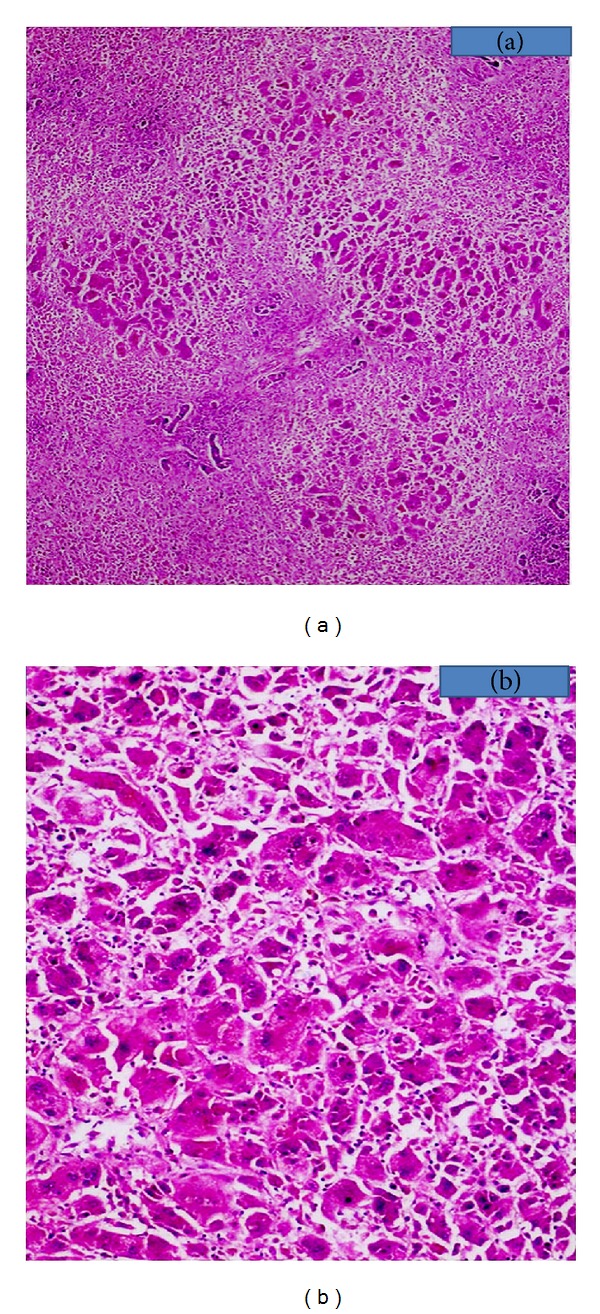
(a) H&E stained section (40x) of explant liver showing massive parenchymal loss and remaining hepatocytes with giant cell transformation. (b) H&E stained section (200x) showing giant cell transformation of hepatocytes.

21-year-old male presented with complaints of fever, myalgia, and arthralgia of one-month duration. Fever was continuous high grade. Myalgia and arthralgia subsided within a week. He had progressive jaundice for 2-3 weeks. On examination, patient had deep icterus, fever, and enlarged liver of 3 cm below right costal margin, and it was tender on palpation. Contrast enhanced computed tomography (CECT) and magnetic resonance imaging (MRI) abdomen showed hepatomegaly, ascites, and bilateral pleural effusion. His serum was positive for anti-HAV IgM, and antinuclear antibody (ANA) titer was 1 : 80. Serum ceruloplasmin and 24-hour urinary copper were normal. His liver function test got worsened within 6 days of hospital course, and his total bilirubin raised from 4.5 to 19.17 mg/dL (normal value 0.2–1 mg/dL), aspartate aminotransferase (AST) raised from 830 to 1490 U/L (normal value 6–40 U/L), alanine aminotransferase (ALT) from 459 to 744 U/L (normal value 4–40 U/L), and international normalized ratio (INR) for prothrombin time raised from 1.5 to 6.2. His serum ammonia level reached 211 microgram/dl, and he developed hepatic encephalopathy. The clinical diagnosis was hepatitis A related acute liver failure. The patient was transplanted according to King's College criteria for acute liver failure, and explant liver on histopathological examination showed submassive necrosis with focal sparing of the portal areas. There was prominent giant cell transformation of viable hepatocytes (Figures 1(a) and 1(b)). Histopathological diagnosis was submassive hepatic necrosis with postinfantile giant cell hepatitis. Patient is on regular followup for 6 months and is doing well. 

## 4. Discussion

PIGCH is very rare in adults (0.1%–0.25% of all hepatic diseases); approximately 100 cases have been reported so far [1–3, 28]. Age and gender do not show any significant preponderance in the series described by Johnson et al., Devaney et al., Phillips et al., Tordjmann et al., and by Micchelli et al. [1–3, 7, 29]. It has been reported from 5 to 80 years of age.

In our institute, total adult liver biopsies were done; only three had postinfantile giant cell hepatitis till date.

Various etiologies associated with postinfantile giant cell hepatitis are summarized in Table 1 and their respective prognosis in Table 2. 

 Medications which can cause PIGCH are methotrexate, 6-mercaptopurine, clometacine, amitriptyline, chlordiazepoxide, p-amino salicylic acid, vinyl chloride, chlorpromazine, herbal medicines, and amoxicillin + clavulanate and doxycycline. These drugs presumed to injure the hepatocytes and cause degenerating effect and formation of giant hepatocytes in certain individuals [1, 3, 5–9]. Most of the reported cases in the literature presented as mild hepatitis [1, 3, 5, 6] except in three; one died due to clometacine induced liver failure [7]; one was a known case of autoimmune hepatitis and was treated with amoxicillin + clavulanate for cellulitis of thigh and clinically deteriorated and required liver transplant [8]. Another case was treated with doxycycline for one week for bacterial bronchitis, and soon he developed acute liver failure and required liver transplant [9].

A variety of autoimmune disorders have been reported as potential cause of PIGCH. It has been reported in cases of autoimmune hepatitis (AIH), systemic lupus erythematosus, autoimmune hemolytic anemia rheumatoid arthritis, primary sclerosing cholangitis, polyarthritis, ulcerative colitis, polyarteritis nodosa, and primary biliary cirrhosis [1, 2, 5, 7, 10–23, 44].

In autoimmune diseases, autoimmune hepatitis (AIH) mainly type I with ANA (ANF) positivity is one of the major cause of PIGCH, accounting for 40% of all autoimmune related cases. The mechanism of giant cell formation in cases of autoimmune disorders is still unknown. Fusion of mononuclear hepatocytes or nuclear proliferation not followed by cell division represents the two prevailing pathogenetic hypotheses [31]. This may be due to autoimmune disease per se or due to both immune complexes, vascular pathology in autoimmune cases creating nutritional challenge to hepatocytes [19]. Clinical course varies from normalization of hepatic histology to progression to cirrhosis and liver failure. The prognosis is dictated by the underlying liver disease. Clinical course is usually severe with most of the patients progressing to rapid onset of cirrhosis [1, 2, 5, 7, 10–23, 31, 44].

Hepatitis A, B, C, E Epstein-Barr virus (EBV), HIV, Cytomegalovirus, and a potentially unidentified paramyxo-like virus have been found to be associated with entity. In a study, human herpes virus 6A infection in a liver transplant recipient was a cause of giant cell hepatitis [1–3, 7, 24–30, 32–34, 36, 37, 45, 46]. 

In HAV infection, PIGCH is a morphological reaction pattern due to the immunoreactivity of viral agents to the hepatocytes. Hepatitis A is an acute infectious disease caused by the hepatitis A virus (HAV), an RNA virus, usually spread by the fecal-oral route. In developing countries and in regions with poor hygiene standards, the incidence of infection with this virus is high. HAV infection produces a self-limited disease that does not result in chronic infection or chronic liver disease. Hepatitis A infection is diagnosed by Anti-HAV IgM antibody. Acute liver failure from Hepatitis A is rare < 0.5%. Hepatitis A infection is diagnosed by Anti-HAV IgM antibody [46]. Four cases of PIGCH were reported out of which one had coexistence of positive ANA. All four had fatal course (acute liver failure) [1, 24–26]. The reported cases had Anti-HAV IgM positivity and ANA positivity had acute fulminant PIGCH, which required an orthotopic liver transplant. Three cases with HBV infection were reported; two had acute hepatitis, and another one had chronic hepatitis, and all three had favorable outcomes [2, 7].

In association to HCV infection, a largest study of 22 biopsies of 18 cases with PIGCH was done by Micchelli et al. Out of these 18 cases, 12 had coinfection with HIV. In addition, there were 2 cases of PIGCH; in HIV/HCV coinfection were also reported. In one patient, there was a progressive clinical worsening after three-month course of prednisone, leading to liver failure and death. His postmortem liver biopsy showed more abundant giant hepatocytes accompanied with the development of a histological pattern of severe fibrosing cholestatic hepatitis. The second patient received a prolonged course of pegylated interferon-alpha-2b and ribavirin with clearance of syncytial giant hepatocytes despite HCV-RNA persistence [30]. Histologically, giant cells were located in zone 3 hepatocytes, were persisted over time, and did not appear to be a marker of aggressive hepatitis [28, 29]. 

In three cases EBV was suggested a possible etiology of giant cell hepatitis resulting in fulminant hepatic failure [31–33].

Paramyxo viral infection including parainfluenza 1, 2, and 3, measles virus, respiratory syncytial virus and distemper virus has been increasingly linked to Postinfantile giant cell hepatitis. Evidence of paramyxo-like viral particles was first reported by Phillips et al. in a series of 10 patients. Five patients' required liver transplantation and the other five died. Other two cases of PIGCH out of which in one patient with CLL presented as an acute hepatitis which lead to cirrhosis in 18 months and other case lead to fulminant course. In these cases also, high-resolution electron micrographs revealed the existence of nucleocapsid-like particles forming aggregates in the cytoplasm of syncytial hepatocytes resembling paramyxo-like viral particles [26, 34, 35]. 

In one patient etiology of PIGCH was suggested as human herpes virus-6A (HHV-6A), who underwent liver transplant for Caroli's disease. He originally had latent infection of human herpes virus-6B (HHV-6B). He developed PICGH at the 13th day of liver transplant. He received organ from a donor with latent infection of human herpes virus-6A. Extensive serologic, molecular and immunohistochemical investigations were done to search for an infectious cause of giant-cell hepatitis. At the onset of the disease, the detection of HHV-6A specific early protein p41/38 in giant cells and later on follow-up samples of plasma, and affected liver tissue suggested that HHV-6A may be a cause of PIGCH. This patient improved clinically, serologically, and histomorphologically after 4 months of treatment [27]. A case of Wilson's disease reported by Welte et al. presented with acute liver failure with presence of syncytial hepatocytes in the liver biopsy. On investigation, this patient was found to be serologically positive for cytomegalovirus [37]. 

Associations with three eosinophilia cases were reported. Two of them had fatal disease course [24, 38], and one had better outcome [32].

Concomitant malignancies have occasionally been described in patients with PIGCH [2]. Two patients with CLL have been reported. A common etiology suggested in both cases was paramyxovirus particles found in giant cells on electron microscopy [34, 39].

Cases with liver transplantation, early recurrence of giant cell hepatitis after liver transplantation favors the hypothesis of a transmissible agent as the etiology of the disease. In a study of seven patients who developed giant cell hepatitis (GCH) after liver transplantation, five of these patients also had GCH as their original liver disease and experienced a particularly aggressive course because of recurrent giant cell hepatitis, beginning 1–21 months after transplantation. Two died and another two required hepatic retransplantation because of recurrent GCH (one of them had GCH recurrence in a second liver allograft). A remaining patient with recurrent GCH is alive for 6 years after transplantation. Followup of the two patients who developed de novo GCH 8 and 24 months after hepatic transplantation showed active micronodular cirrhosis. All of these cases were serologically negative for hepatitis viruses. None had a history of drug exposure. Two patients had an associated autoimmune syndrome, which could have been the cause of GCH. Human papilloma virus (HPV) type 6 was detected in liver tissues with GCH from one of three cases before and three of four cases after transplantation. Recurrent disease in five of seven patients suggested that this entity may be related to a transmissible agent or that a particular recipient may injure liver in a way that elicits a giant cell reaction [40]. Routine follow-up liver biopsy is necessary in these cases in order to gain more information about the precise incidence and aggressively of disease recurrence in the allograft [38, 40–42].

Postinfantile giant cell hepatitis clinical spectrum varies from acute hepatitis to mild chronic liver in the form of icteric disease, [7, 29] to rapid progression of cirrhosis, and to subacute hepatic failure to fatal hepatic failure [1–40, 44–47]. Adult giant-cell hepatitis has been shown to be progressive and often fatal disease process, with a survival rate of only approximately 50% without orthotopic liver transplantation [3, 5, 7, 26, 29, 38–42]. The high mortality rate is often due to severe liver failure, or sepsis in the setting of aggressive use of immunosuppressant [3, 5, 7, 26, 29, 38–42].

Raised bilirubin, slightly raised to markedly raised transaminases autoantibody markers are positive in around 50% cases mostly ANA/ANF [1, 5, 7, 10–23, 44]. In other cases, viral markers are positive where etiology viral [1–3, 5, 7, 24–30, 32–37, 39, 45, 46].

Gross examination liver biopsy may be of uniformly dark green to grayish brown in color [3]. Liver is usually shrunken, but in some acute cases it can be enlarged [3]. Microscopically, diagnostic giant cells are the common pathological finding. Other biopsy findings are periportal lymphocytic infiltrate (T lymphocytes), massive necrosis, bridging necrosis, “activated” perisinusoidal cells, bilirubinostasis, and Mallory hyaline bodies, often associated with neutrophilic infiltrate and severe fibrosis [1–3, 7, 29]. 

In most cases, the giant cell change found more than two-thirds of the parenchyma. Giant cell transformation is most pronounced in zone 3. The giant cells often contain 4 to 20 centrally allocated nuclei [1] (Figures 1(a) and 1(b)). In cases of HAV, Paramyxo virus, EBV, few autoimmune related cases, and hypereosinophilia related and post transplant HPV-6 related cases show giant cell predominance in periportal periseptal areas with muliti-acinar necrosis and increased inflammation [1, 3, 23–26, 32–35, 38–42]. Cases showed some degree of periportal fibrosis to severe fibrosis [1, 2, 5, 7, 29]. Progression to rapid onset of cirrhosis was evident in the biopsy specimens in cases particularly with autoimmune diseases [24–27, 30, 32–37, 39, 45, 46], and submassive to massive necrosis of liver parenchyma were seen in cases with HAV, paramyxo virus infection, those with positive EBV serology, hypereosinophilia, and post transplant recurrence of PIGCH [1, 3, 24–26, 32–35, 38–41].

The mechanisms by which the characteristic multinucleated hepatocytes syncytia formed are unknown. Two processes have been proposed: increased hepatocytes nuclear proliferation that is not followed by cell division or the membrane fusion of neighboring hepatocytes [2, 13, 15, 44, 48]. In adults, giant cell change of hepatocytes represents an unusual and idiosyncratic regenerative response to a wide variety of hepatic stimuli [4, 48, 49].

There is no established treatment for paramyxo virus induced PIGCH. There are a few sporadic case reports in the literature where ribavirin treatment was successful but failed in another cases [41]. This drug, which has been shown to be quite effective against paramyxo virus, needs further clinical evaluation for this particular viral cause related to PIGCH [39, 48–51].

In PIGCH in HCV-HIV coinfection and isolated HCV positive cases, specific treatment with pegylated interferon and ribavirin can lead to histological resolution and biochemical improvement, even in the absence of HCV-RNA clearance [11]. A considerable number of patients exhibit autoimmune features and they respond to prednisone therapy alone or in combination with immunosuppressant such as Azathioprine as recommended by AASLD 2002 (American Association for the Study of Liver Diseases) [5, 12–22, 44, 52]. In posttransplant cases of PIGCH cyclophosphamide therapy claimed to be life saving and effective by few [51].

## 5. Conclusion

Autoimmune causes account for approximately 40% of PIGCH, which commonly presents as chronic liver disease while 25% of cases can have an acute presentation and few of them have rapid onset of cirrhosis [2, 4, 12–22, 28, 44]. Autoimmune diseases with presentation of giant cell hepatitis have moderate sort of prognosis [12–22, 44]. Those with paramyxo virus, EBV, HAV, and post transplant HPV induced PIGCH have subfulminant to fulminant course and required orthotopic liver transplant [1, 3, 24–26, 33–35, 38–42]. In HCV, HBV, HEV, HCV-HIV induced PIGCH have relatively better outcome [5, 7, 28, 29]. Overall PIGCH presents clinically as severe form of hepatitis [53].

## Figures and Tables

**Table 1:** Various etiological agents of post infantile giant cell hepatitis.

Drugs and medication	Methotrexate, clometacin, 6-mercaptapurine, p-aminosalicylic acid, vinyl chloride, amitriptyline, chlordiazepoxide, and chlorpromazine and herbal medicine

Autoimmune diseases	Systemic lupus erythematosus, rheumatoid arthritis, polyarthritis, ulcerative colitis, autoimmune hemolytic anemia, primary sclerosing cholangitis, and autoimmune hepatitis (AIH), polyarteritis nodosa, and primary biliary cirrhosis

Viral causes	Hepatitis A, B, C, E Epstein-Barr virus (EBV), HIV, paramyxo-like virus. herpesvirus 6A infection, and human papillomavirus.

Miscellaneous	Hypereosinophilia, chronic lymphocytic leukaemia, lymphoma, sarcoidosis, Kugelberg-Welander syndrome, hypoparathyroidism, Sickle cell anaemia, and post transplant

**Table 2:** Cases of post infantile Giant hepatitis with their prognostic outcome.

Etiology	Number of cases	Prognostic outcome	References
Drugs			
Methotrexate	2	Good (mild hepatitis)	[1, 5]
Chlorpromazine	1	Good (mild hepatitis)	[1]
ISABGOL	1	Good (mild hepatitis)	[6]
Clometacine	1	Poor (acute liver failure)	[7]
Amoxicillin and Clavulanate	1	Poor (chronic hepatitis with acute decompensation)	[8]
Doxycycline	1	Poor (acute liver failure) Treated for a week for bacterial bronchitis	[9]

Autoimmune			
AIH	5	1 (died) 4 Good (clinically improved)	[1]
	2	1 Moderate (rapid onset of cirrhosis died) 1 Moderate (rapid onset of cirrhosis)	[5]
	10	Moderate 25% (acute hepatitis), 42% moderate (chronic active hepatitis), 33% moderate to poor cirrhosis, >1-month duration	[2]
AIH	13	4 Poor (liver failure)	[7]
		5 Moderate (rapid cirrhosis)	
		4 Good (responded to immunosuppressants)	
	1	Moderate (rapid onset of cirrhosis)	[10]
	1	Good (responded to immunossuppresion)	[11]
SLE	2	Moderate	[2, 12]
Autoimmune hemolytic anemia	3	Poor	[13–15]
PSC + AIH	2	Moderate (rapid onset of cirrhosis) Good (mild hepatitis)	[16, 17]
AIH + polyarthritis	1	Moderate (early cirrhosis)	[18]
AIH + polyarteritis	1	Moderate (early cirrhosis)	[19]
AIH + UC	1	Moderate (early cirrhosis)	[20]
PBC	2	1 Poor, (liver failure) 1 moderate (early cirrhosis)	[21, 22]
AIH II	1	Poor (died)	[23]

Viral			
HAV	4	Poor (fatal liver failure)	[1, 24–26]
HEV	1	Good (mild hepatitis)	[27]
HBV	3	Good (1 acute hepatitis, 2 chronic hepatitis)	[2, 7]
HCV	22	Good (chronic hepatitis)	[5, 28–30]
EBV	3	Poor (fatal liver failure)	[31–33]
Paramyxoviruses	13	Poor (7 fatal liver failure, 6 died)	[3, 26, 34, 35]
HIV + HCV	2	Good (chronic hepatitis)	[30]
HIV	2	Good (chronic hepatitis)	[36]
HHV-6A	1	Good (chronic hepatitis)	[27]
CMV	1	Poor (acute liver failure with underlying Wilson's diseases)	[37]

Hypereosinophilia	3	2 Poor (liver failure) 1 Good	[24, 32, 38]
CLL	3	2 Poor (liver failure) 1 Good (responded to immunosuppression)	[39]
Posttransplant	10	Poor (recurrent disease, mostly required retransplant)	[40–43]
